# Genome Sequences of 12 *Pseudomonas lundensis* Strains Isolated from the Lungs of Humans

**DOI:** 10.1128/genomeA.01461-17

**Published:** 2018-02-15

**Authors:** Brittan S. Scales, John R. Erb-Downward, Nicole R. Falkowski, John J. LiPuma, Gary B. Huffnagle

**Affiliations:** a Division of Pulmonary and Critical Care Medicine, Department of Internal Medicine, University of Michigan Medical School, Ann Arbor, Michigan, USA; b Department of Microbiology and Immunology, University of Michigan Medical School, Ann Arbor, Michigan, USA; c Department of Pediatrics and Communicable Diseases, University of Michigan Medical School, Ann Arbor, Michigan, USA; d Department of Molecular, Cellular and Developmental Biology, University of Michigan Medical School, Ann Arbor, Michigan, USA; e Mary H. Weiser Food Allergy Center, University of Michigan Medical School, Ann Arbor, Michigan, USA

## Abstract

We report here the first complete genome sequence of a human *Pseudomonas lundensis* isolate, strain AU1044, and the draft genomes of 11 other clinical *P. lundensis* strains, isolated from the lungs of cystic fibrosis patients. The genome of strain AU1044 is 4.81 Mb and encodes seven 16S rRNAs.

## GENOME ANNOUNCEMENT

Due to genetic diversity among strains, the *Pseudomonas fluorescens* species complex is comprised of multiple subgroups/subclades (1–5). In 2015, our group published the first draft genomes of clinical strains within the *P. fluorescens* species complex (6). Based on phylogeny by Loper et al. (2), the draft genomes of the clinical strains were assigned to subclades 1, 2, and 3 of the *P. fluorescens* species complex, but additional phylogenetic analysis of the *P. fluorescens* species complex by Garrido-Sanz et al. (4, 5) suggested that these clinical strains could be assigned to the *P. protegens*, *P. koreensis*, and *P. fluorescens* subgroups, respectively. In either case, the clinical strains aligned with reported subgroups/subclades built using previously sequenced environmental strains.

Here, we report 12 clinical isolates from the *P. fluorescens* species complex that were identified as *P. lundensis* based on multilocus sequence analysis (MLSA). Analysis of the 16S rRNA gene in our 12 clinical isolates indicates >99.8% identity with *P. lundensis* DSM6252^T^. *P. lundensis* is a psychrotrophic bacterium that contributes to cold food spoilage. Prior to this study, the only sequenced genomes of this species had been isolated from spoiled meat.

The 12 *P. lundensis* strains were each isolated from the sputum of a different cystic fibrosis patient. Initial isolation of strains occurred between 1 February 1999 and 30 June 2006 from eight treatment centers across the United States (Ann Arbor, MI; Boston, MA; Chapel Hill, NC; Cincinnati, OH; Columbia, MO; Hartford, CT; Palo Alto, CA; and St. Louis, MO). Storage of isolates and sequencing of genomes were performed as previously reported (6). Isolates were banked at −80°C. PCR amplification of the 16S rRNA gene from individual colonies was performed using the universal primer set 8F and 1492R (7). The amplified genes were sequenced and screened against the NCBI nucleotide database and the EzBioCloud 16S database. The isolates, identified as *P. lundensis*, were grown aerobically overnight in Luria broth at 34°C. Genomic DNA was isolated with the Qiagen DNeasy blood and tissue kit (catalog no. 69506). Sequence data were generated from a 100-bp paired-end library on the Illumina HiSeq 2000 platform. The sequence reads were assembled *de novo* using DNAstar SeqMan NGen version 12 software into an average of 63.8 contigs (range, 35 to 113). The Mauve aligner was used to reorder the contigs (8). The assembled draft genomes contained an average G+C content of 58.9% (range, 57.7% to 61.13%) and had an average genomic size of 4.9 Mb (range, 4.8 to 5.4 Mb). Strain AU1044 was further sequenced on two PacBio P4-C2 single-molecule real-time cells, and reads were assembled into a closed genome with the in-house procedure at the Genomics Resource Center at the University of Maryland. AU1044’s closed genome has a G+C content of 57.7% and is 4.92 Mb in size. MLSA was performed using *dnaE*, *ppsA*, *recA*, *rpoB*, *guaA*, *mutL*, *pyrC*, and *acsA*, modified from Loper et al. (2). Clustering and creation of the phylogenetic tree was completed with MAFFT (9, 10).

### Accession number(s).

The sequences have been deposited in DDBJ/ENA/GenBank under the accession numbers listed in Table 1.

**TABLE 1:** Genome sequence data of the 12 reported *P. lundensis* strains

GenBank accession no.	Strain	No. of contigs	Genome size (Mb)	G+C content (%)	Assembly status
CP017687	AU1044	1	4.88	57.9	Whole
LCYS00000000	AU2390	93	5.41	58.3	Draft
LCYT00000000	AU7350	57	4.85	58.7	Draft
MLCS00000000	AU9518	113	5.22	57.8	Draft
LCYU00000000	AU10414	56	4.84	58.6	Draft
LCYV00000000	AU11122	58	4.84	58.6	Draft
LCYY00000000	AU11136	61	4.84	58.6	Draft
LCYW00000000	AU11164	56	4.83	58.7	Draft
LCYX00000000	AU11235	61	4.84	58.7	Draft
LCYZ00000000	AU12597	35	4.9	58.8	Draft
LCZA00000000	AU12644	62	4.87	58.8	Draft
MLCU00000000	AU14541	99	5.23	57.6	Draft
